# A Case–Control Study of Prenatal Thallium Exposure and Low Birth Weight in China

**DOI:** 10.1289/ehp.1409202

**Published:** 2015-05-22

**Authors:** Wei Xia, Xiaofu Du, Tongzhang Zheng, Bin Zhang, Yuanyuan Li, Bryan A. Bassig, Aifen Zhou, Youjie Wang, Chao Xiong, Zhengkuan Li, Yuanxiang Yao, Jie Hu, Yanqiu Zhou, Juan Liu, Weiyan Xue, Yue Ma, Xinyun Pan, Yang Peng, Shunqing Xu

**Affiliations:** 1 Key Laboratory of Environment and Health, Ministry of Education & Ministry of Environmental Protection, and State Key Laboratory of Environmental Health, School of Public Health, Tongji Medical College, Huazhong University of Science and Technology, Wuhan, Hubei, People’s Republic of China; 2 Zhejiang Provincial Center for Disease Control and Prevention, Zhejiang, People’s Republic of China; 3 Department of Epidemiology, Brown University, Providence, Rhode Island, USA; 4 Women and Children Medical and Healthcare Center of Wuhan, Wuhan, Hubei, People’s Republic of China; 5 Department of Environmental Health Sciences, Yale School of Public Health, New Haven, Connecticut, USA; 6 Macheng Maternity and Child Health Care Hospital, Macheng, Hubei, People’s Republic of China; 7 Ezhou Maternal and Child Health Hospital, Ezhou, Hubei, People’s Republic of China

## Abstract

**Background:**

Thallium (Tl) is a highly toxic heavy metal widely present in the environment. Case reports have suggested that maternal exposure to high levels of Tl during pregnancy is associated with low birth weight (LBW), but epidemiological data are limited.

**Objectives:**

This study was designed to evaluate whether prenatal Tl exposure is associated with an increased risk of LBW.

**Methods:**

This case–control study involving 816 study participants (204 LBW cases and 612 matched controls) was conducted in Hubei Province, China, in 2012–2014. Tl concentrations were measured in maternal urine collected at delivery, and associations with LBW were evaluated using conditional logistic regression.

**Results:**

Higher maternal urinary Tl levels were significantly associated with increased risk of LBW [crude odds ratio (OR) = 1.52; 95% CI: 1.00, 2.30 for the highest vs. lowest tertile], and the association was similarly elevated after adjustment for potential confounders (adjusted OR = 1.90; 95% CI: 1.01, 3.58 for the highest vs. lowest tertile). Stratified analyses showed slightly higher risk estimates for LBW associated with higher Tl levels for mothers < 28 years old and for mothers with lower household income; however, there was no statistical evidence of heterogeneity in risk according to maternal age (*p* for heterogeneity = 0.18) or household income (*p* for heterogeneity = 0.28).

**Conclusion:**

To our knowledge, ours is the first case–control study to investigate the association between prenatal Tl exposure and LBW. The results suggest that prenatal exposure to high levels of Tl may be associated with an increased risk of LBW.

**Citation:**

Xia W, Du X, Zheng T, Zhang B, Li Y, Bassig BA, Zhou A, Wang Y, Xiong C, Li Z, Yao Y, Hu J, Zhou Y, Liu J, Xue W, Ma Y, Pan X, Peng Y, Xu S. 2016. A case–control study of prenatal thallium exposure and low birth weight in China. Environ Health Perspect 124:164–169; http://dx.doi.org/10.1289/ehp.1409202

## Introduction

Thallium (Tl), a well-known toxic heavy metal, is present naturally in the earth’s crust and is now widely used in the manufacturing of optical lenses, semiconductors, scintillation counters, low-temperature thermometers, chemical catalysts, crystals, and imitation jewelry (Rodríguez-Mercado and Altamirano-Lozano 2013). Tl is released into the environment from a variety of anthropogenic sources, such as mining activities, coal and oil combustion, cement plants, and refining processes (Kazantzis 2000). China is the largest producer and consumer of Tl in the world, and, as a result, there is increased concern about the widespread presence of this metal in the environment (Liu et al. 2010; Xiao et al. 2012; Yang et al. 2005).

Increased exposure to Tl in industrial workers and in the general population has raised concerns for human health (Peter and Viraraghavan 2005). Of particular interest are possible adverse outcomes associated with prenatal exposure to Tl, because fetuses are known to be more susceptible to some chemical exposures than adults (Stillerman et al. 2008). High-level Tl exposures, during pregnancy, due to industrial pollution have been associated with increased risk of congenital abnormalities (Dolgner et al. 1983). Previous case reports suggested that Tl poisoning occurring during pregnancy is associated with fetal death, prematurity, or decreased birth weight (Hoffman 2000).

Low birth weight (LBW), which is defined as a birth weight of < 2,500 g, is a main cause of morbidity and mortality in the neonatal period, and is one of the main risk factors of global disease burden, according to the World Health Organization (WHO 2002). Previous research has found an association between LBW and a variety of adverse health outcomes during childhood, some of which may persist into adulthood (Gluckman et al. 2008). Prenatal exposure to heavy metals such as arsenic, cadmium, and lead has been associated with decreased birth weight (Hopenhayn et al. 2003; Ronco et al. 2009; Zhu et al. 2010). Except for a small pilot study that reported a negative association between maternal blood Tl concentration and birth weight in 81 Chinese mother–infant pairs (Hu et al. 2015), we are not aware of any epidemiological studies that have investigated the association between prenatal exposure to Tl and risk of LBW. As a follow-up to the report by Hu et al. (2015), we conducted a case–control study to evaluate the association between prenatal Tl exposure and the risk of LBW among 816 pregnant women (including 204 cases and 612 matched controls) in Hubei province, China.

## Methods

*Study design and study population.* A nested case–control study design was used in this study. Both cases and controls were selected from the prospective Tongji birth cohort, which was conducted to explore the environmental and genetic factors that affect health and development. This ongoing cohort enrolls participants at three maternity hospitals in the cities of Wuhan, Ezhou, and Macheng, which are located in Hubei province in the central region of People’s Republic of China. The recruitment period started in November 2012 and will continue for 3 years. Pregnant women, who came for their first examination in the first trimester or gave birth at one of the three hospitals, have been asked to participate in the study. The eligibility criteria for participants are as follows: *a*) residence in the study areas at the time of the recruitment period with an expectation to reside continually in these areas for the foreseeable future, and *b*) ability to comprehend the Chinese language and complete the questionnaire. Participants were invited to provide blood and urine samples and participate in a face-to-face interview. Also, the participants will be contacted by telephone for follow-up. The participating children will be followed through the use of questionnaires and health examinations at 1 month, 6 months, 1 year, 3 years, 6 years, and 12 years of age. Between November 2012 and April 2014, 16,293 women were recruited from three hospitals (9,209 from Wuhan, 4,550 from Ezhou, and 2,534 from Macheng), and the participation rate (number of participants/number of potentially eligible women) was 78.7%. The research protocol was approved by the ethics committee of the Tongji Medical College, Huazhong University of Science and Technology, and the three study hospitals. All participants provided written informed consent at enrollment.

In this study, cases were mothers who delivered a singleton live infant with birth weight < 2,500 g in one of the three hospitals. Controls were mothers who delivered a singleton live infant with normal birth weight between ≥ 2,500 g and < 4,000 g. Women with multiple pregnancies, without urine samples available for analysis, and those who gave birth to a stillborn infant or an infant with a birth defect were excluded. For each case, three individual controls were randomly selected from the birth cohort based on the matching variables by delivery hospital, infant sex, and maternal age at conception (within 1-year interval). If more than three potential controls met the matching criteria for an individual case, the three women whose maternal ages were closest to that of the case mother were selected. Controls were sampled from the cohort without replacement, so an individual control could be matched to one case only. A total of 204 cases and 612 controls were included in the analysis.

*Data collection.* The face-to-face interviews were conducted by specially trained nurses in the hospitals with the participants after delivery. The interview collected a variety of information, including socioeconomic data (e.g., maternal age, education, occupation, household income, and self-reported weight before pregnancy) and lifestyle factors during pregnancy (e.g., smoking, passive smoking, and alcohol consumption). Information about the mothers’ history of pregnancy outcomes, disease, and information concerning the infants’ birth date, sex, gestational age at birth, and birth weight were retrieved from medical records. Gestational age was estimated using the date of the last menstrual period. Birth weight of infants was measured within 1 hr after birth by experienced obstetric nurses using standardized procedures. The body mass index (BMI) of mothers was calculated using the self-reported weight before pregnancy and height, which was measured using a stadiometer.

*Urine sample collection and Tl measurement.* The maternal urine samples were obtained during admission to the hospital as part of the preparation for delivery (within 3 days before delivery). All of the urine samples were collected in polypropylene tubes, and stored at –20°C until further analysis.

Before analysis, urine samples were thawed at room temperature, and 1 mL of urine from the supernatant was introduced in 15-mL Kirgen polypropylene conical centrifuge tubes. Next, 3% HNO_3_ was added to the final volume of 5 mL for overnight nitrification. The resulting sample was digested by ultrasound at 40°C for 1 hr and then analyzed for Tl by inductively coupled plasma mass spectrometry (ICP-MS) (Agilent 7700; Agilent Technologies). The operation conditions of ICP-MS were RF power 1550 W, plasma gas flow 15.00 L/min, auxiliary gas flow 0.9 L/min, carrier gas flow 0.25 L/min, resolution (peak high 10%) 0.65 ~ 0.80 amu, improved quantity of samples 0.4 mL/min, unimodal residence time 0.1 sec.

All maternal urinary Tl levels were analyzed without knowledge of case–control status. The Tl measurements were repeated three times and the average was used for all statistical analyses. The Standard Reference Material Human Urine (SRM2670a Toxic Elements in Urine; National Institute of Standards and Technology, Gaithersburg, MD, USA) was used as an external quality control in each batch to assess the instrument performance, and the concentrations measured were within the certified range recommended by the manufacturer (5%). If concentrations were significantly different from the certified value of SRM2670a, the instrument was recalibrated and the previous batch of samples was reanalyzed. A 3% HNO_3_ blank was processed in each batch of samples to control for possible contamination. The limit of detection (LOD) for Tl was 0.02 μg/L. The recovery of the quality control standard by using this procedure was 103%. The intra-day coefficient of variation (CV) was 1.34%, and inter-day CV was 2.07%. The undetected samples (*n* = 5) were assigned a value of one-half the LOD. Lead, arsenic, and cadmium were also measured simultaneously, because previous studies have suggested that they are potentially associated with decreasing birth weight (Hopenhayn et al. 2003; Ronco et al. 2009; Zhu et al. 2010). The recoveries of the three heavy metals varied from 85% to 100%, and the intra-day and inter-day CV was within 5%. The detection rate of lead, arsenic, and cadmium in maternal urine samples was 98.2%, 100%, and 99.8%, respectively.

Urine creatinine concentrations were determined by a creatinine kit (Mindray BS-200 CREA Kit; Shenzhen Mindray Bio-medical Electronics Co., Ltd). Tl concentrations in urine (micrograms per liter) were adjusted for creatinine to account for variations in urine dilution in spot urine specimens, and results were expressed as μg/g creatinine.

*Statistical analysis.* The distributions of Tl concentrations were tested by the Shapiro–Wilk normality test. Because Tl concentrations were highly skewed, the Wilcoxon matched pairs signed rank test was used to compare Tl concentrations between cases and controls. The Pearson chi-square test was used to evaluate the differences in the variables between cases and controls. The associations between the risk of LBW and maternal urinary Tl levels were evaluated by calculating matched odds ratios (OR) and 95% confidence intervals (CIs) using conditional logistic regression models. Models were fit using maternal urinary Tl concentrations as categorical variables, based on the tertile distribution of Tl concentrations in controls, and the lowest tertile was assigned as the referent group. In this study, the three variables that may represent socioeconomic status (SES) including household income (≥ 50,000 or < 50,000 yuan per year), maternal education (more than high school, high school, less than high school), and occupational status (employed or unemployed), were weakly correlated (Pearson correlation coefficients were *r* = 0.28 between income and education, and *r* = 0.12 between income and occupation). The likelihood ratio test was used to assess model fit, and inclusion of all three SES variables (income, education, occupation) into the model did not significantly improve the model fit compared to addition of each individual variable into the model separately. We selected household income to adjust for SES in this study because its adjustment showed a larger impact on the ORs for the association between Tl and LBW than did the other two SES variables. Although gestational age (< 37 or ≥ 37 weeks), parity (1 or ≥ 2), maternal BMI (underweight, normal, overweight), passive smoking (yes or no), and hypertension during pregnancy (yes or no) individually did not cause a change in the ORs by ≥ 10%, inclusion of all of these variables in the model together did result in a > 10% change in the ORs for Tl, and thus they were all included in the final model. The missing values were constructed as dummy variables in the regression model. We did not adjust for maternal smoking or alcohol consumption during pregnancy because these behaviors were reported by only one case (for smoking) and by two cases and one control (for alcohol consumption) (Table 1).

**Table 1:** Basic characteristics of LBW cases and controls [*n* (%)].

Characteristic	Cases (*n *= 204)	Controls (*n *= 612)	*p-*Value
Delivery hospital			NA
Wuhan	158 (77.4)	474 (77.4)
Ezhou	22 (10.8)	66 (10.8)
Macheng	24 (11.8)	72 (11.8)
Infant sex			NA
Male	101 (49.5)	303 (49.5)
Female	103 (50.5)	309 (50.5)
Maternal age (years)			NA
< 25	48 (23.5)	146 (23.8)
25–29	81 (39.7)	242 (39.5)
30–34	58 (28.4)	174 (28.4)
≥ 35	17 (8.3)	50 (8.2)
Education			< 0.01
More than high school	77 (37.8)	322 (52.6)
High school	38 (18.6)	120 (19.6)
Less than high school	89 (43.6)	167 (27.3)
Missing	0 (0.0)	3 (0.5)
Occupation			0.17
Employed	152 (74.5)	491 (80.2)
Unemployed	44 (21.6)	97 (15.9)
Missing	8 (3.9)	24 (3.9)
Household income (yuan per year)			< 0.01
≥ 50,000	61 (29.9)	279 (45.6)
< 50,000	116 (56.9)	275 (44.9)
Missing	27 (13.2)	58 (9.5)
BMI (kg/m^2^)			0.02
Normal (18.5–23.9)	108 (52.9)	385 (62.9)
Underweight (< 18.5)	60 (29.4)	125 (20.4)
Overweight (≥ 24)	26 (12.8)	85 (13.9)
Missing	10 (4.9)	17 (2.8)
Smoking during pregnancy			0.02
No	198 (97.1)	608 (99.4)
Yes	1 (0.5)	0 (0.0)
Missing	5 (2.4)	4 (0.6)
Passive smoking during pregnancy			0.69
No	149 (73.0)	464 (75.8)
Yes	47 (23.0)	129 (21.1)
Missing	8 (3.9)	19 (3.1)
Alcohol use during pregnancy			0.31
No	196 (96.1)	594 (97.1)
Yes	2 (1.0)	1 (3.3)
Missing	6 (2.9)	17 (2.8)
Parity			0.22
1	159 (77.9)	501 (81.9)
≥ 2	45 (22.1)	111 (18.1)
Hypertension during pregnancy			< 0.01
No	183 (89.7)	598 (97.7)
Yes	20 (9.8)	12 (2.0)
Missing	1 (0.5)	2 (0.3)
Gestational age (weeks)			< 0.01
> 37	96 (47.1)	598 (97.7)
≤ 37	108 (52.9)	14 (2.3)
NA, not applicable (matching factor).			

We tested for linear trends by modeling the median values of tertiles of Tl as a continuous variable and evaluated the statistical significance of this predictor using the Wald test. The analyses were further stratified by infant sex and maternal age. The median age of the case mothers at delivery (28 years old) was used as the cut-point for these stratified analyses, regardless of the age of the matched controls (one matched control mother with an age of 29 years was distributed into the ≤ 28 year old stratum). We also performed sensitivity analyses that excluded maternal urine samples with creatinine < 0.3 g/L or > 3 g/L (WHO 1996), preterm births (gestational age < 37 weeks), and hypertension during pregnancy. In the sensitivity analyses, the entire matched set was excluded if the case or any one of the three matched controls had these conditions. To evaluate the association between maternal urinary Tl levels and LBW stratified by education, occupation, and household income, we used unconditional logistic regression models; these were adjusted for the matched factors (delivery site, maternal age, and infant sex) and other potential confounders. In addition, to evaluate potential confounding by lead, cadmium, and arsenic on LBW, we also performed a conditional multivariable logistic regression analysis that included the potential confounders and the heavy metals measured in maternal urine.

All statistical analyses were performed using SAS (version 9.3; SAS Institute Inc.). All statistical tests were considered to be significant at an alpha level of 0.05 for a two-tailed test.

## Results

Table 1 presents general characteristics of the 204 cases and 612 controls. Approximately 77% of the participating mothers were enrolled from Wuhan, 11% were from Ezhou, and 12% were from Macheng. There were 101 sets of boys and 103 sets of girls. The mean age of all the participating mothers was 28.1 ± 4.7 years old. Compared with the controls, the case mothers had lower educational attainment and lower household income, and were more likely to be underweight. There were a significantly higher proportion of case mothers who had hypertension during pregnancy and who had preterm birth compared with the controls. The average gestational weeks of infants in the cases and controls were 36.2 ± 2.3 and 38.9 ± 1.2, respectively. We also found that the LBW cases were significantly associated with lower household income, low BMI, hypertension during pregnancy, and gestational age in the multivariable model (see Supplemental Material, Table S1).

The detection rate for Tl in maternal urine was 99.4%. The creatinine-adjusted Tl concentrations ranged from below the LOD to 8.15 μg/g creatinine with a median of 0.64 μg/g creatinine in the mothers of LBW cases, and from below the LOD to 6.90 μg/g creatinine with a median of 0.55 μg/g creatinine in the control mothers (see Supplemental Material, Table S1). There was no significant difference in the median Tl concentrations between the two groups (*p* > 0.05). Also, there were no significant differences in the Tl concentrations for different educational levels, occupational status, household income, or for other demographic characteristics.

The unadjusted and adjusted ORs and 95% CIs for LBW according to the tertiles of creatinine-adjusted Tl concentrations in maternal urine are shown in Table 2. Compared with the lowest tertile of urinary Tl concentrations, a significant trend was found between LBW risk and increasing levels of Tl in the unadjusted analysis (OR = 1.19; 95% CI: 0.79, 1.78 for the medium tertile and OR = 1.52; 95% CI: 1.00, 2.30 for the highest tertile) (*p* trend = 0.06). After adjustment for potential confounders, the positive association between LBW risk and maternal urinary Tl levels was similarly elevated with a significant trend (adjusted OR = 1.61; 95% CI: 0.89, 2.91 for the medium tertile and 1.90; 95% CI: 1.01, 3.58 for the highest tertile) (*p* trend = 0.04). When 7 cases and 18 controls with creatinine concentrations < 0.3 g/L or > 3.0 g/L and their matched cases or controls were excluded (180 case–control sets included), we found the adjusted OR for LBW was essentially unchanged (adjusted OR = 1.88; 95% CI: 0.98, 3.63 for the highest vs. lowest tertile). In the analysis excluding preterm births (87 case/control sets included), an adjusted OR of 1.81 (95% CI: 0.90, 3.67) was observed for the highest tertile, which was slightly decreased due to the smaller sample size. We also performed an analysis that excluded the women with hypertension during pregnancy (178 case–control sets included), and found that the adjusted OR for LBW associated with Tl exposure was essentially unchanged (adjusted OR = 1.97; 95% CI: 1.04, 3.75 for the highest vs. lowest tertile).

**Table 2:** Association between maternal urinary thallium levels and LBW.

Thallium (μg/g creatinine)	Cases/controls (*n*)	OR^*a*^ (95% CI)	OR^*b*^ (95% CI)
Total (*n *= 816)
< 0.39	56/204	1.00	1.00
0.39–0.77	67/204	1.19 (0.79, 1.78)	1.61 (0.89, 2.91)
≥ 0.78	81/204	1.52 (1.00, 2.30)	1.90 (1.01, 3.58)
*p* for trend^*c*^		0.06	0.04
Excluding urine creatinine < 0.3 or > 3 g/L (*n *= 720)
< 0.39	53/180	1.00	1.00
0.39–0.74	53/180	0.99 (0.64, 1.53)	1.40 (0.74, 2.64)
≥ 0.75	74/180	1.49 (0.96, 2.30)	1.88 (0.98, 3.63)
*p* for trend^*c*^		0.01	0.07
Excluding preterm birth (*n *= 348)
< 0.38	23/87	1.00	1.00
0.38–0.78	31/87	1.36 (0.73, 2.52)	1.39 (0.63, 2.58)
≥ 0.79	33/87	1.52 (0.78, 2.97)	1.81 (0.90, 3.67)
*p* for trend^*c*^		0.29	0.15
Excluding hypertension (*n *= 712)
< 0.39	49/178	1.00	1.00
0.39–0.77	55/178	1.11 (0.72–1.73)	1.59 (0.86–2.98)
≥ 0.78	74/178	1.62 (1.04–2.54)	1.97 (1.04–3.75)
*p* for trend^*c*^		0.03	0.04
The unadjusted and adjusted estimates were derived using conditional logistic regression to account for matching on delivery hospital, infant sex, and maternal age at conception (within 1 year). ^***a***^Unadjusted odds ratio. ^***b***^Adjusted for gestational age, household income, maternal BMI, parity, passive smoking, and hypertension during pregnancy. ^***c***^*p*-Values for trend were derived using a continuous variable with the median value of each tertile.			

Table 3 shows results stratified by maternal age and infant sex. The adjusted OR for the highest tertile of Tl levels was 2.46 (95% CI: 1.04, 5.88) for LBW in mothers < 28 years old, and 2.09 (95% CI: 0.86, 5.27) in mothers ≥ 28 years old (*p* heterogeneity = 0.18). Associations were similar for male (adjusted OR = 1.88; 95% CI: 0.72, 4.96 for the highest vs. lowest tertile) and female infants (OR = 1.90; 95% CI: 0.79, 4.65) (*p* heterogeneity = 0.68).

**Table 3:** Association between maternal urinary thallium levels and low birth weight, stratified by maternal age and infant sex.

Thallium (μg/g creatinine)	Cases/controls (*n*)	OR^*a*^ (95% CI)	OR^*b*^ (95% CI)	*p* for heterogeneity
Maternal age (years)				0.18
< 28 (*n *= 392)
< 0.38	31/98	1.00	1.00
0.38–0.73	24/98	0.74 (0.40, 1.37)	0.76 (0.39, 1.84)
≥ 0.74	43/98	1.59 (0.88, 2.86)	2.46 (1.05, 5.88)
*p* for trend^*c*^		0.06	0.04
≥ 28 (*n *= 424)
< 0.41	25/106	1.00	1.00
0.41–0.78	40/106	1.58 (0.90, 2.79)	1.93 (0.84, 5.11)
≥ 0.79	41/106	1.66 (0.93, 2.96)	2.09 (0.86, 5.27)
*p* for trend^*c*^		0.15	0.37
Infant sex				0.68
Male (*n *= 404)
< 0.42	27/101	1.00	1.00
0.42–0.79	36/101	1.30 (0.74, 2.29)	1.24 (0.51, 3.02)
≥ 0.80	38/101	1.44 (0.79, 2.61)	1.88 (0.72, 4.96)
*p* for trend^*c*^		0.27	0.20
Female (*n *= 412)
< 0.39	29/103	1.00	1.00
0.39–0.72	30/103	1.05 (0.58, 1.89)	1.57 (0.72, 4.01)
≥ 0.73	44/103	1.63 (0.91, 2.92)	1.90 (0.79, 4.65)
*p* for trend^*c*^		0.07	0.14
The unadjusted and adjusted estimates were derived using conditional logistic regression to account for matching on delivery hospital, infant sex, and maternal age at conception (within 1 year). ^***a***^Unadjusted odds ratio. ^***b***^Adjusted for gestational age, household income, maternal BMI, parity, passive smoking, and hypertension during pregnancy. ^***c***^*p*-Values for trend were derived using a continuous variable with the median value of each tertile.				

We also performed unconditional analyses stratified by household income, maternal educational level, and occupational status, because of the differences between cases and controls in these variables (see Supplemental Material, Table S2). The highest tertile of Tl was positively associated with LBW among mothers who had lower household income (adjusted OR = 2.53; 95% CI: 1.13, 5.99 compared with the lowest tertile), but not in those with higher household incomes (OR = 1.19; 95% CI: 0.37, 2.17). However, the differences between household income strata were not significant (*p* for heterogeneity = 0.28). Also, there was no statistical evidence of heterogeneity in risk according to educational levels (*p* for heterogeneity = 0.62) or occupational status (*p* for heterogeneity = 0.89).

We further performed a conditional multivariable logistic regression analysis with all variables, including the potential confounders and the heavy metals (Tl, lead, arsenic, and cadmium). The associations between higher levels of Tl and increased risk of LBW were largely unchanged (adjusted OR = 1.91; 95% CI: 0.95, 4.01 for the highest vs. lowest tertile) (see Supplemental Material, Table S3).

## Discussion

To the best of our knowledge, this is the first case–control study to investigate the association of prenatal Tl exposure with the risk of LBW. The results of the present study suggest that prenatal exposure to the current levels of Tl encountered today in China may potentially increase the risk of delivering LBW infants.

Tl was detected in almost all of the maternal urine samples, indicating that our study population is widely exposed to this heavy metal in daily life. A comparison of urinary Tl concentrations in pregnant women of the present study and previously published data from non-occupationally exposed populations worldwide is shown in Table 4. The pregnant women in our study had higher levels of urinary Tl (arithmetic mean, median, and geometric mean of 0.40, 0.32, and 0.28 μg/L, respectively) compared with the general populations in developed countries, such as in the United States [geometric mean, 0.16 μg/L (Navas-Acien et al. 2005); median, 0.15 μg/L (Yorita Christensen 2013)], and Germany [arithmetic mean, 0.15 μg/L; geometric mean, 0.07 μg/L (Heitland and Köster 2006)]. Compared with pregnant women in previous studies, our study population also had higher levels than those reported in pregnant women from the United States [geometric mean, 0.17 μg/L (Jain 2013)], and Spain [arithmetic mean, 0.18 μg/g creatinine compared with 0.40 μg/g creatinine in our study population (Fort et al. 2014)]. There are currently limited data on Tl exposure levels in the general population in China, and no previous study has reported the urinary Tl levels in Chinese pregnant women. A study conducted in Guiyang city, China, detected urinary Tl levels in four people who were non-occupationally exposed [arithmetic mean, 0.65 μg/L (Xiao et al. 2007)], which was similar to the levels observed in our study. Maternal urinary Tl levels in our study population were lower than levels in 503 women in Magu village [arithmetic mean, 2.43 μg/g creatinine (Zhang et al. 2011)], which is located near a Tl mining area in Guizhou Province, China, that has been reported to have relatively high environmental levels of Tl (Xiao et al. 2004). In China, as a result of rapid economic growth, Tl emissions from industrial sources including mineral extraction and processing have been increasing rapidly (Liu et al. 2010; Xiao et al. 2012). Due to the current widespread use of Tl, the potential health effects of Tl exposure in the general population require more attention.

**Table 4:** Comparison of thallium concentrations in urine from the present study and previous studies.

Reference	Location	Sampling years	*n*	Population	Arithmetic mean	Median	Geometric mean
Present study	Hubei, China	2012–2014	816	Pregnant women	0.40 μg/L (0.89 μg/g creatinine)	0.32 μg/L (0.56 μg/g creatinine)	0.28 μg/L (0.59 μg/g creatinine)
Xiao et al. 2007	Guiyang, China	2002	4	General population	0.65 μg/L	—	—
Zhang et al. 2011	Magu, China	2010	503	General population	2.43 μg/g creatinine	—	—
Navas-Acien et al. 2005	USA	1999–2000	2,465	General population	—	—	0.16 μg/L
Yorita Christensen 2013	USA	2007–2008	1,587	General population	—	0.15 μg/L	—
Jain 2013	USA	2003–2010	1,565	Pregnant women	—	—	0.17 μg/L
Fort et al. 2014	Spain	2004–2006	657	Pregnant women	0.18 μg/g creatinine	< LOD	—
Heitland and Köster 2006	Germany	2005	87	General population	0.15 μg/L	—	0.07 μg/L
—, not reported.							

A previous study showed that the children born in an industrial area with Tl contamination had higher levels of urinary Tl (mean, 5.2 μg/L), and had about a 6-fold higher risk of congenital abnormalities compared with the unexposed children (Dolgner et al. 1983). Hoffman (2000) reported that pregnant women exposed to high levels of Tl (urinary Tl > 120 μg/L) gave birth to premature and LBW infants. In the present study, we provide evidence for an association between higher level of maternal urinary Tl and an increased risk of LBW, and the association was unchanged after excluding the samples with the creatinine concentrations < 0.3 g/L or > 3.0 g/L and hypertension during pregnancy. Infants born preterm account for about two-thirds of all LBW infants (Martin et al. 2013), and consistent with this, our sample size was reduced to only 87 sets of cases and controls after preterm births were excluded. However, associations between LBW and Tl were comparable to the total population estimates when the analysis was restricted to term births. In addition, the association between higher levels of Tl and increased risk of LBW was not affected by adjustment for lead, arsenic, and cadmium. Exposure to these metals has been associated with decreasing birth weight in previous studies (Hopenhayn et al. 2003; Ronco et al. 2009; Zhu et al. 2010). Our findings are consistent with a pilot study by Hu et al. (2015) of 81 mother–infant pairs in four Chinese cities, which reported that Tl levels were associated with decreased infant birth weight.

In the stratified analysis, we found that the significant association between maternal urinary Tl levels and the risk of delivering a LBW infant was slightly more pronounced in younger mothers (< 28 years old) compared with older mothers (≥ 28 years old) and in the mothers who had lower household income compared with those who had higher household income, although no significant effect modification was apparent. Previous studies have reported that younger mothers and mothers with lower household income generally displayed greater risk of delivering a LBW infant (Bradley and Corwyn 2002), and a reason might be that they are less likely to have access to prenatal care.

Although there is currently limited understanding of the potential mechanism between maternal exposure to Tl and an increased risk of LBW infants, one possible mechanism is that Tl may exert toxicity by disturbing mitochondrial function (Bragadin et al. 2003; Korotkov and Lapin 2003). Tl crosses the placenta freely (Hoffman 2000) and may affect mitochondrial function in placenta and fetal tissue, which is critical for embryonic and fetal development and growth (Lane et al. 1998). Also, some evidence suggests that Tl triggers oxidative stress in the cell through increasing lipid oxidation (Hanzel and Verstraeten 2006) and inhibits enzymes with active sites containing cysteine residues (Mulkey and Oehme 1993). Increased oxidative stress may also play an important role in restricting fetal growth (Mert et al. 2012); thus, Tl-induced oxidative stress may also impair fetal growth.

One of the strengths of our study is that the nested case–control design provided the opportunity to include all the LBW infants in the study. In addition, interviews conducted with all participants allowed us to adjust for other potential risk factors for LBW, such as gestational age, maternal BMI, household income, passive smoking, and hypertension during pregnancy.

There are some limitations to this study. First, maternal urinary Tl levels were measured at only one spot time before delivery and may not be perfect surrogates for prenatal Tl levels, though urinary Tl is regarded to represent a steady-state condition with long-term exposure in the general population (Xiao et al. 2007). A prospective study of Tl concentrations at multiple points may help to evaluate whether there is a critical exposure window of Tl on fetal development, and clarify if the change in mothers’ physiological parameters (e.g., hypertension) over the course of the pregnancy affects urinary Tl levels. Second, maternal nutritional status was not addressed in this study, but low maternal levels of nutrients are known to adversely affect fetal development.

## Conclusion

Using a case–control study design, we found a positive association between maternal urinary Tl and LBW in Chinese women. Additional research is needed to confirm the association between prenatal exposure to Tl and LBW and develop strategies for reducing LBW related to developmental exposure to environmental pollutants, including Tl.

Editor’s Note: The author lineup changed between advance and final publication. All authors of the article agreed to the changes. The correct author lineup is included in this article.

## Supplemental Material

(323 KB) PDF
