# Supplementary material for: A Case–Control Study of Prenatal Thallium Exposure and Low Birth Weight in China
Source: Environ Health Perspect. 2015 May 22;124(1):164–9. doi: 10.1289/ehp.1409202 (PMC4710601; doi:10.1289/ehp.1409202)
Supplement: (323 KB) PDF [file ehp.1409202.s001.acco.pdf]

Environ Health Perspect
DOI: 10.1289/ehp.1409202

Note to Readers: *EHP* strives to ensure that all journal content is accessible to all readers. However, some figures and Supplemental Material published in *EHP* articles may not conform to 508 standards due to the complexity of the information being presented. If you need assistance accessing journal content, please contact ehp508@niehs.nih.gov. Our staff will work with you to assess and meet your accessibility needs within 3 working days.

Supplemental Material

A Case–Control Study of Prenatal Thallium Exposure and Low Birth Weight in China

Wei Xia, Xiaofu Du, Bin Zhang, Yuanyuan Li, Bryan A. Bassig, Aifen Zhou, Youjie Wang, Chao Xiong, Zhengkuan Li, Yuanxiang Yao, Jie Hu, Yanqiu Zhou, Qi Wang, Juan Liu, Weiyan Xue, Yue Ma, Xinyun Pan, Yang Peng, Tongzhang Zheng, and Shunqing Xu

Table of Contents

Table S1. The distribution of maternal urinary thallium concentrations (µg/g creatinine), and the association between the variables and risk of low birth weight.

Table S2. Association between maternal urinary thallium levels and low birth weight, stratified by maternal education, occupational status, and household income.

Table S3. Association between maternal urinary thallium levels and low birth weight adjusted by other heavy metals.

Table S1. The distribution of maternal urinary thallium concentrations (μg/g creatinine), and the association between the variables and risk of low birth weight.

Characteristics	Tl concentration in cases			Tl concentration in controls			OR[a] (95%CI)	OR[b] (95%CI)
	n	Median	Range	n	Median	Range		
**All participants**	204	0.64	<LOD-8.15	612	0.55	<LOD-6.90	——	——
**Delivery hospital**							NA	NA
Wuhan	158	0.61	<LOD-8.15	474	0.52	<LOD-6.90		
Ezhou	22	0.95	0.12-5.46	66	0.87	0.25-6.03		
Macheng	24	0.52	0.15-2.02	72	0.51	0.05-2.54		
**Infant gender**							NA	NA
Male	101	0.65	<LOD-8.15	303	0.59	<LOD-6.90		
Female	103	0.63	0.04-5.46	309	0.52	<LOD-6.68		
**Maternal age**							NA	NA
<25	48	0.67	0.13-5.46	146	0.57	0.04-6.23		
25-29	81	0.63	0.02-2.87	242	0.52	<LOD-5.49		
30-34	58	0.68	<LOD-8.15	174	0.56	0.08-6.90		
≥35	17	0.50	0.04-1.79	50	0.57	<LOD-6.19		
**Education**								
More than high school	77	0.57	<LOD-8.15	322	0.50	<LOD-6.90	1.00	1.00
High school	38	0.69	0.04-2.87	120	0.58	0.14-6.23	1.54 (0.97, 2.45)	1.44 (0.73, 2.86)
Less than high school	89	0.68	0.04-5.46	167	0.59	0.05-6.03	3.54 (2.23, 5.62)	1.68 (0.82, 3.44)
Missing	0	——	——	3	0.58	0.58	——	——
**Occupational status**								
Employed	152	0.60	<LOD-8.15	491	0.54	<LOD-6.90	1.00	1.00
Un-employed	44	0.71	0.08-2.44	97	0.58	0.15-8.19	1.50 (0.99, 2.26)	0.84 (0.44, 1.62)
Missing	8	0.52	0.15-5.46	24	0.66	0.29-3.15		
**Household income**								
≥50,000 yuan per year	61	0.59	0.04-4.88	279	0.57	<LOD-6.68	1.00	1.00
<50,000 yuan per year	116	0.66	<LOD-8.15	275	0.52	<LOD-6.90	2.11 (1.45, 3.06)	1.84 (1.05, 3.23)
Missing	27	0.68	0.15-5.46	58	0.70	0.09-6.18	——	——
**Body mass index**								
Normal (18.5-23.9)	108	0.61	<LOD-8.15	385	0.56	<LOD-6.90	1.00	1.00
Underweight (<18.5)	60	0.60	0.40-4.88	125	0.57	<LOD-6.23	1.72 (1.18, 2.51)	2.57 (1.48, 4.44)

Characteristics	Tl concentration in cases			Tl concentration in controls			OR[a] (95%CI)	OR[b] (95%CI)
	n	Median	Range	n	Median	Range		
Overweight (≥24)	26	0.68	0.13-2.28	85	0.50	0.09-4.70	1.05 (0.65, 1.71)	0.75 (0.36, 1.57)
Missing	10	0.74	0.14-5.46	17	0.70	0.26-3.15	——	——
**Smoking during pregnancy**								
No	198	0.63	<LOD-8.15	608	0.55	<LOD-6.90	——	——
Yes	1	0.48	0.48	0	——	——	——	——
Missing	5	0.86	0.68-5.46	4	0.59	0.12-1.07	——	——
**Passive smoking during pregnancy**								
No	149	0.63	<LOD-8.15	464	0.53	<LOD-6.90	1.00	1.00
Yes	47	0.72	0.02-3.94	129	0.62	0.06-5.49	1.12 (0.78, 1.63)	0.76 (0.36, 1.22)
Missing	8	0.58	0.17-5.46	19	0.55	0.09-4.49	——	——
**Alcohol use during pregnancy**								
No	196	0.64	<LOD-8.15	594	0.54	<LOD-6.90	——	——
Yes	2	0.59	0.48-0.72	1	0.67	0.67	——	——
Missing	6	0.82	0.43-5.46	17	0.98	0.13-5.06	——	——
**Parity**								
1	159	0.59	<LOD-8.15	501	0.55	<LOD-6.90	1.00	1.00
≥2	45	0.69	0.04-2.44	111	0.57	0.12-6.19	1.38 (0.88, 2.17)	1.06 (0.51, 2.22)
**Hypertension during pregnancy**								
No	184	0.65	<LOD-8.15	600	0.55	<LOD-6.90	1.00	1.00
Yes	20	0.6	0.15-2.70	12	0.54	<0.20-1.90	7.45 (3.12, 17.81)	9.11 (2.34, 35.53)
Missing	1	0.86	0.86	2	2.02	1.77-2.27	——	——
**Gestational age**								
≥37 weeks	96	0.61	0.07-8.15	598	0.54	<LOD-6.90	1.00	1.00
<37 weeks	108	0.67	<LOD-3.94	14	0.67	0.12-1.67	34.92 (17.68, 68.96)	44.35 (20.48, 96.07)

Abbreviations: Tl, thallium; LOD, limit of detection; OR, odds ratio; CI, confidence interval; NA, not applicable (matching factor).

[a] Unadjusted odds ratio.

[b] Adjusted odds ratio. Obtained from the multivariable conditional logistic regression model that simultaneously included all the listed factors and the maternal thallium levels.

Table S2. Association between maternal urinary thallium levels and low birth weight, stratified by maternal education, occupational status, and household income.

Thallium (μg/g creatinine)	Cases/ Controls	OR[a] (95% CI)	OR[b] (95% CI)	*p* for heterogeneity
**Education**				0.62
**Less than high school (n=256)**				
<0.43	27/56	1.00	1.00	
0.43-1.06	37/56	1.37 (0.74, 2.55)	1.30 (0.56, 2.98)	
≥ 1.07	25/55	0.94 (0.49, 1.82)	0.95 (0.45, 2.39)	
p for trend [c]		0.66	0.10	
**High School (n=158)**				
<0.43	11/40	1.00	1.00	
0.43-0.74	11/40	1.00 (0.39, 2.57)	1.22 (0.33-4.53)	
≥0.75	16/40	1.46 (0.60, 3.52)	1.51 (0.47-5.44)	
p for trend [c]		0.28	0.54	
**Higher than high school (n=399)**				
<0.38	19/108	1.00	1.00	
0.38-0.72	28/107	1.48 (0.78, 2.80)	1.31 (0.86, 2.95)	
≥0.73	30/107	1.59 (0.85, 3.00)	1.76 (0.90, 5.35)	
p for trend [c]		0.56	0.19	
**Household income**				0.28
**< 50,000 yuan per year (n=391)**				
<0.38	30/92	1.00	1.00	
0.38-0.68	31/92	1.03 (0.58, 1.84)	1.67 (0.70, 3.96)	
≥0.69	55/91	1.85 (1.09, 3.15)	2.53 (1.13, 5.99)	
p for trend [c]		0.05	0.02	
**≥ 50,000 yuan per year (n=340)**				
<0.40	17/93	1.00	1.00	
0.40-0.76	23/93	1.35 (0.68, 2.70)	1.27 (0.44, 2.40)	
≥0.77	21/93	1.24 (0.61, 2.49)	1.19 (0.37, 2.17)	
p for trend [c]		0.65	0.66	
**Occupational status**				0.89
**Un-employed (n=141)**				
<0.39	12/33	1.00	1.00	
0.39-0.76	13/32	1.12 (0.44, 2.81)	1.22 (0.28, 5.32)	
≥0.77	19/32	1.63 (0.68, 3.90)	1.86 (0.46, 7.27)	
p for trend [c]		0.89	0.32	
**Employed (n=643)**				
<0.43	40/164	1.00	1.00	
0.43-0.77	52/164	1.30 (0.82, 2.07)	1.49 (0.82, 2.74)	
≥0.78	60/163	1.51 (0.96, 2.38)	1.58 (0.87, 2.88)	
p for trend [c]		0.16	0.34	

Abbreviations: OR, odds ratio; CI, confidence interval.

[a] Unadjusted odds ratio. [b] Adjusted for delivery site, maternal age, infant gender, gestational age, maternal body mass index, parity, passive smoking, and hypertension during pregnancy. The unadjusted and adjusted estimates were derived using conditional logistic regression to account for matching on delivery hospital, infant gender, and maternal age at conception (within 1 year). [c] *p*-values for trend were derived using continuous variable with the median value of each tertile.

Table S3. Association between maternal urinary thallium levels and low birth weight adjusted by other heavy metals.

**Metal (μg/g creatinine)**	**Cases**	**Controls**	**OR[a] (95% CI)**	**OR[b] (95% CI)**	**OR[c] (95% CI)**
**Thallium**					
<0.39	56	204	1.00	1.00	1.00
0.39-0.77	67	204	1.19 (0.79, 1.78)	1.61 (0.89, 2.91)	1.50 (0.81, 2.80)
≥0.78	81	204	1.52 (1.00, 2.30)	1.90 (1.01, 3.58)	1.91 (0.95, 4.01)
p trend [c]			0.06	0.04	0.07
**Cadmium**					
<0.34	41	204	1.00	1.00	1.00
0.34-0.74	76	204	1.91 (1.24, 2.94)	1.89 (1.02, 3.51)	1.62 (0.84, 3.15)
≥0.75	87	204	2.40 (1.53, 3.78)	1.57 (0.81, 3.03)	1.22 (0.55, 2.71)
p trend [c]			< 0.01	0.52	0.95
**Lead**					
<5.25	50	204	1.00	1.00	1.00
5.25-11.80	71	204	1.51 (0.99, 2.32)	1.45 (0.79, 2.66)	1.23 (0.64, 2.34)
≥11.81	83	204	1.84 (1.19, 2.87)	1.25 (0.65, 2.43)	0.91 (0.41, 1.98)
p trend [c]			0.01	0.72	0.88
**Arsenic**					
<16.11	65	204	1.00	1.00	1.00
16.11-26.76	74	204	1.13 (0.77, 1.67)	1.35 (0.78, 2.34)	1.21 (0.68, 2.16)
≥26.77	65	204	1.00 (0.67, 1.51)	1.15 (0.63, 2.07)	0.97 (0.50, 1.90)
p trend [c]			0.85	0.88	0.65

Abbreviations: OR, odds ratio; CI, confidence interval.

[a] Unadjusted odds ratio. [b] Adjusted for gestational age, maternal body mass index, household income, parity, passive smoking, and hypertension during pregnancy. [c] Adjusted for the listed heavy metals, gestational age, maternal body mass index, household income, parity, passive smoking, and hypertension during pregnancy. The unadjusted and adjusted estimates were derived using conditional logistic regression to account for matching on delivery hospital, infant gender, and maternal age at conception (within 1 year). [c] *p*-values for trend were derived using continuous variable with the median value of each tertile.
